# Influence of the Nb and V Addition on the Microstructure and Corrosion Resistance of the Fe-B-Co-Si Alloy

**DOI:** 10.3390/ma14144045

**Published:** 2021-07-20

**Authors:** Rafał Mech, Jolanta Gąsiorek, Amadeusz Łaszcz, Bartosz Babiarczuk

**Affiliations:** Faculty of Mechanical Engineering, Wroclaw University of Science and Technology, 50-370 Wroclaw, Poland; jolanta.gasiorek@pwr.edu.pl (J.G.); amadeusz.laszcz@pwr.edu.pl (A.Ł.); bartosz.babiarczuk@pwr.edu.pl (B.B.)

**Keywords:** corrosion resistance, iron-based alloy, microstructure, energy harvesting

## Abstract

The paper presents a comparison of the results of the corrosion resistance for three Fe-B-Co-Si-based newly developed alloys with the addition of Nb and V. The corrosion performance differences and microstructure variations were systematically studied using scanning electron microscope, electric corrosion equipment, X-ray diffractometer, and differential calorimeter. It has been shown that each alloying addition increased the corrosion resistance. The highest corrosion resistance obtained by potentiodynamic polarization was found for the alloy with both Nb and V addons (Fe_57_Co_10_B_20_Si_5_Nb_4_V_4_) and lowest in the case of the basic four-element Fe_62_Co_15_B_14_Si_9_ material. This shows that the proper choice of additions is of significant influence on the final performance of the alloy and allows tailoring of the material for specific applications.

## 1. Introduction

The energy harvesting (EH) term was introduced to common use a few years ago and it is still of high interest in many fields of science and the industry. This is mainly due to the fact that more and more devices surrounding us demand energy. EH is usually described as a set of methods that allow obtaining electricity from different physical phenomena, such as mechanical, thermal, solar, electromagnetic energy, salinity gradients, etc. Vibrations are an excellent example of mechanical energy that can be converted to electricity. Research results show that the development of EH from vibrations can be useful in solving many practical problems, such as providing energy for wireless sensor networks [1,2,3], self-powered sensors [4], structural health monitoring [5], medical devices (pacemakers) [6], etc. EH might also be an interesting alternative to commonly used batteries and, even in the near future, allow the development of maintenance-free self-powered devices. Nowadays, the energy that might be received from vibration power harvesting is still a few times lower than the one that is cumulated in traditional batteries. However, the ongoing development of electrical circuits dedicated to low power solutions (CMOS circuits and VLSI design) significantly reduced the power consumption of commercial wireless sensors from mW to μW [7], which provides more opportunities for the use of vibration energy harvesting in real applications such as self-powered single and multi-node wireless sensors nets. Furthermore, developing new devices based on vibration harvesting energy technology provides a possibility to use these devices in areas of limited accessibility, such as the human body (implants), civil engineering structures, or mechanical engineering structures (devices embedded in the structure).

In the case of vibration energy harvesting technology, there are four distinguished mechanisms which are usually developed by the researchers: electromagnetic [8], electrostatic [9], piezoelectric [10], and magnetostrictive [11]. The use of piezoelectric and magnetostrictive mechanisms is strictly connected with the properties of related materials, which belong to the group of smart materials. Energy harvesters based on piezoelectric materials convert mechanical energy into electrical energy by the direct piezoelectric effect [12]. Nowadays, the most popular material used for vibration energy harvesting is a piezoelectric material mainly because of the compact configuration of the devices, reasonable electro-mechanical coupling coefficient, and good compatibility with MEMS. There are many scientific papers in which research on piezoelectric energy harvesters was described [13,14,15,16,17]. In [13] an arc-shaped piezoelectric vibration energy harvester was proposed. Paper [14] shows a system based on the flow-induced vibration of a piezoelectric composite cantilever pipe. Additionally, a method allowing the measurement of the energy harvesting efficiency was shown. An attractive solution was presented in [15] where a novel design of a piezoelectric sheet rotational energy harvesting system as an alternative to cantilever beams. Additionally, in [16] a rotational energy harvester allowed a high voltage generation at low rpm. Promising results were also obtained in [17] where the temperature sensor was powered with the use of only a piezoelectric harvester in the form of a flag without the necessity of storing energy. Energy harvesting based on piezoelectric effect allows the production of high output voltage and low current, which in many solutions cause that devices to be powered only for a short period. Additionally, some piezoelectric materials cannot withstand large deflections and are brittle.

Opposing piezoelectric material harvesters based on magnetostrictive materials can produce higher power density at low frequencies of work. Magnetostrictive materials are metals in pure form or metal alloys. Interest in these materials as potential energy converters in vibration energy harvesting is rising in recent years. Harvesters based on magnetostrictive materials use the Villari effect and Faraday electromagnetic induction effect. The load in the form of vibration causes deformation of the material, which changes the magnetization of the material. This change of magnetization can be transformed into electricity using pick-up coils placed around magnetostrictive materials. In comparison to piezoelectric-based harvesters, the magnetostrictive-based devices do not age, do not require external voltage or charge source, and do not depolarize.

Currently, the most commonly used magnetostrictive material for energy harvesting devices based on the Villari effect is an intermetallic alloy of Td, Dy, and Fe elements, commercially known as Terfenol-D [18]. This material has a high magneto-mechanical coupling coefficient and high energy density. However, due to the drawbacks such as brittleness, size, and shape caused by the complicated production process (elements are in the shape of a cylinder or cuboid [19]), its use in energy harvesting solutions is debatable. Nevertheless, researchers are still working on overcoming these disadvantages. One of the solutions is the development of modified materials by adding new alloying elements or preparing composite structures.

While in the case of modifying the composition of the metallic alloy, no significant improvement in properties was achieved [20,21] and in the case of composite materials with various matrices it was possible to reduce the brittleness and increase the range of operating frequency, while at the same time slightly decreasing the value of obtained magnetostriction. Most of the works devoted to composites focused on the use of various types of matrices, such as epoxy [22,23], phenolic [22,24], thermoplastic [25], or vinyl esters [26]. Other possible types of composites are composites based on metallic matrices [27,28]. The work carried out so far has focused mainly on using forged matrices made of aluminum or copper. The authors of this work took steps to produce composite materials consisting of a powder of magnetostrictive material and a soft magnetic material with a nanocrystalline structure. Research works devoted to magnetostrictive composites are mainly focused on determining the magnetomechanical properties of the obtained materials and often ignored the fact of their later use. Although, as previously noted, it is believed that the devices using energy recovery from vibrations will soon find more applications and that the working environment will be diversified, the main emphasis in this work was on the analysis of the influence of alloy additives in developed materials and on their soft magnetic properties and corrosion resistance.

In this study, three types of soft magnetic materials were fabricated: Fe_62_Co_15_B_14_Si_9_, Fe_67_Co_10_B_12_Si_9_Nb_2_, and Fe_57_Co_10_B_20_Si_5_Nb_4_V_4_. Structural studies of the produced samples were then carried out using X-ray diffraction (XRD), differential scanning calorimetry (DSC), and scanning electron microscopy SEM to determine the crystallographic structure of the fabricated samples. The main focus is on corrosion testing. The electrochemical impedance spectroscopy (EIS) and potentiodynamic polarization measurements were discussed in detail to obtain information about the corrosion resistivity of the investigated samples.

## 2. Materials and Methods

Metallic alloy compositions presented in this work were developed based on the papers devoted to well-known soft magnetic amorphous material commercially known as FINEMET [29,30]. There exist research describing a modification of Finemet-type alloys. However, most of them are based on similar to the original amount of composition elements, with minor modifications. Materials presented in this work have a significantly lower amount of Fe and Co in their compositions in comparison to Finemet-type alloys. Compositions are distinguished below:

- Fe_62_Co_15_B_14_Si_9_;
- Fe_67_Co_10_B_12_Si_9_Nb_2_;
- Fe_57_Co_10_B_20_Si_5_Nb_4_V_4_.

The alloys presented in this paper are described using atomic notation.

**Figure 1:**
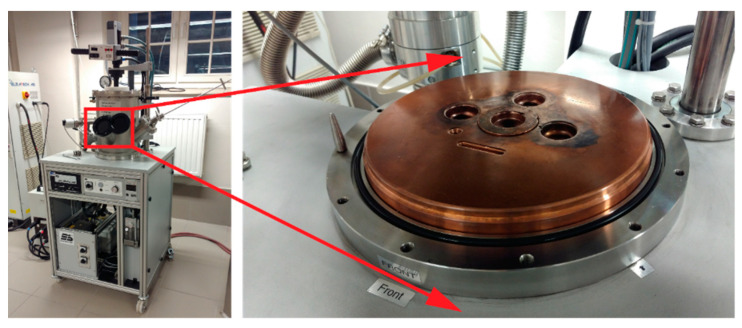
Laboratory arc furnace (arc-melter) from Edmund Bühler GmbH. In the middle of the copper plate is a suction casting mold and around it are milled recesses for remelting the elements.

Alloying elements were melted into a uniform homogeneous material with the use of a laboratory arc furnace (arc-melter) by Edmund Bühler GmbH (Bodelshausen, Germany). In order to obtain the highest accuracy of mapping the chemical composition, elements of very high purity (from 99.5% to 99.999%) provided by Alfa Aesar (Thermo Fisher GmbH, Kandel, Germany) in the form of irregular pieces, granules, or wires were used to produce the designed materials. Each element was weighed to an accuracy of ±0.0002 g and placed in a sterile container. The alloying elements prepared in this manner were mixed and placed in the furnace to obtain an alloy material. In order to avoid oxidation and contamination of the prepared alloys, before starting the melting procedure, the furnace is pumped down to pressure (5 × 10^−2^ Pa) five times and filled with argon to atmospheric pressure. Then, using a diffusion pump, the furnace chamber is pumped down to high vacuum conditions (5 × 10^−5^ Pa) and refilled with argon to a pressure of 600–800 mbar. Finally, an electric arc with a melting current of 150–250 A is used to melt the chosen chemical elements. Melting takes place on a copper water-cooled disc with milled recesses for individual alloys (Figure 1). Due to the cohesive forces between the liquid and metallic elements, the material obtained as a result of such melting is in the form of a metallic pastille. In order to obtain the most homogeneous material structure and to allow diffusion processes to occur in the entire volume of the prepared alloy, each of the pastilles is melted three times and turned between each individual remelting.

Next, from the previously prepared metallic alloys in the form of pastilles, thin rods are made by the suction casting method by using the Edmund Bühler GmbH furnace installation. The prepared pastilles were placed in a special copper mold and mounted in the furnace system (Figure 1). As in the case of the previous step, the procedure of melting the material and its vacuum suction was preceded by pumping the furnace chamber to the pressure (5 × 10^−2^ Pa) five times and filling it with argon to atmospheric pressure in each cycle. Then, by using a diffusion pump, the furnace chamber and the vacuum suction tanks are pumped down to high vacuum conditions (5 × 10^−5^ Pa). The furnace chamber is refilled with argon to a pressure of 600–800 mbar, while the previously obtained vacuum is left in the vacuum suction tanks to allow the liquid melt to be sucked in later.

**Figure 2:**
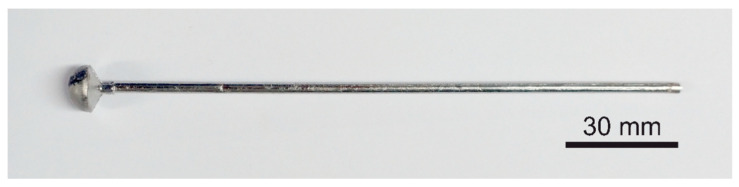
The rod obtained by the method of suction casting.

An electric arc with a melting current of 150–250 A is used to melt the prepared materials in the form of pastilles. When the alloy is liquid in all its volume, the valve connecting the furnace chamber with the vacuum tanks is opened. The pressure difference causes the material to be immediately sucked into the constantly water-cooled mold. The materials produced by this method are in the form of a rod with a diameter of 3 mm and a length of 150 mm (Figure 2).

The prepared three types of specimens were subjected to tests to determine their microstructure. For this purpose, the Ultima IV X-ray diffractometer (Rigaku, Tokyo, Japan), the Netzsch STA449 F1 differential calorimeter (NETZSCH Analysing & Testing, Selb, Germany) and the S-3400N (Hitachi, Tokyo, Japan) scanning electron microscope (SEM) were used.

Finally, detailed tests of the corrosion resistance of the manufactured samples with different chemical compositions of Fe_62_Co_15_B_14_Si_9_, Fe_67_Co_10_B_12_Si_9_Nb_2_, and Fe_57_Co_10_B_20_Si_5_Nb_4_V_4_ were carried out. Three test samples of each produced alloy were used in the corrosion resistance studies. The samples prepared for corrosion tests were ground using 400, 600, 800, 1000, 1200, 1500, and 2000 grit sanding paper and subsequently polished with 6 µm, 3 µm, and 1 µm diamond abrasive suspension. Three-electrode system, platinum electrode as auxiliary, saturated calomel electrode (SCE) as reference, and a sample as work electrode were used to measure the EIS and potentiodynamic polarization in 0.5 M NaCl solution at room temperature condition (25 °C) by the ATLAS 1131&EU potentiostat (Atlas Solich, Rębiechowo, Poland). The open-circuit potential (OCP) was recorded by 2 h—until the changes were lower than 5 mV/5 min. The potentiodynamic polarization was measured in the −300 mV to +300 mV potential range with respect to the OCP vs. SCE with a scan rate of 1 mV/s. The polarization resistance (R_p_) was calculated based on the corrosion current densities (j_corr_) and cathodic and anodic Tafel’s slopes (B_c_/B_a_) by using Equation (1).

(1) $$R_{p}=\frac{\mathrm{Ba}\;*\;\mathrm{Bc}}{2.3\left(\left|\mathrm{Ba}\right|+\left|\mathrm{Bc}\right|\right)}\frac{1}{\mathrm{jcorr}}=\frac{B}{\mathrm{Jcorr}},$$

The EIS measurement was conducted in a frequency range from 10 mHz to 100 kHz with amplitude 1 mV and 8 points/dec. The AtlasLab software (AtlasSollich, Rębiechowo, Poland) was used to fit EIS data. For each set of samples, 3 repetitions were performed. The measured area was 1 cm^2^ and the area was cleaned used ethanol (96%, POCH, Gliwice, Poland) before the test. Additionally, after the corrosion tests, the samples were again examined under the SEM to determine the corrosion products.

## 3. Results

**Figure 3:**
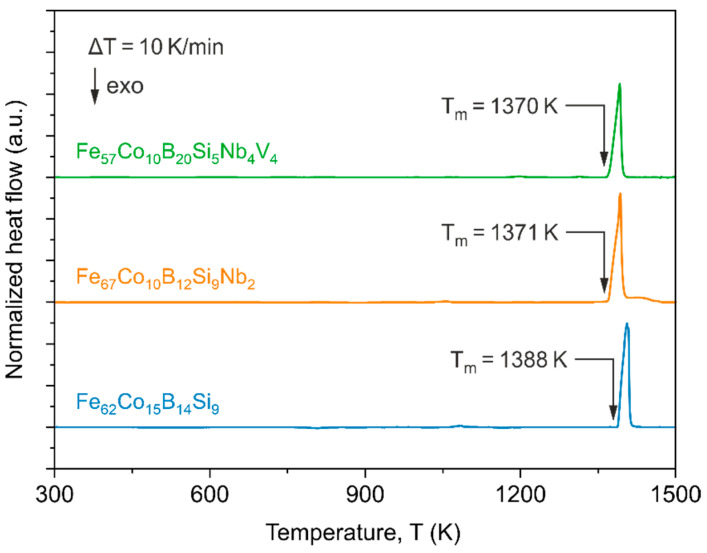
DSC curves for the Fe_62_Co_15_B_14_Si_9_, Fe_67_Co_10_B_12_Si_9_Nb_2_, and Fe_57_Co_10_B_20_Si_5_Nb_4_V_4_ alloys.

The DSC curves of as-quenched alloys are presented in Figure 3. No significant changes that could indicate the first and second-order phase transition characteristic for metallic alloys with an amorphous structure are noticed in all three alloys. Single endothermic peaks marked at the end of each curve allow us to determine the melting point T_m_ of the alloys. The highest T_m_ = 1388 K is received for the Fe_62_Co_15_B_14_Si_9_ alloy and T_m_ temperatures for the other two alloys were similar and are equal to 1371 K and 1370 K for the Fe_67_Co_10_B_12_Si_9_Nb_2_ and Fe_57_Co_10_B_20_Si_5_Nb_4_V_4_ alloys, respectively.

**Figure 4:**
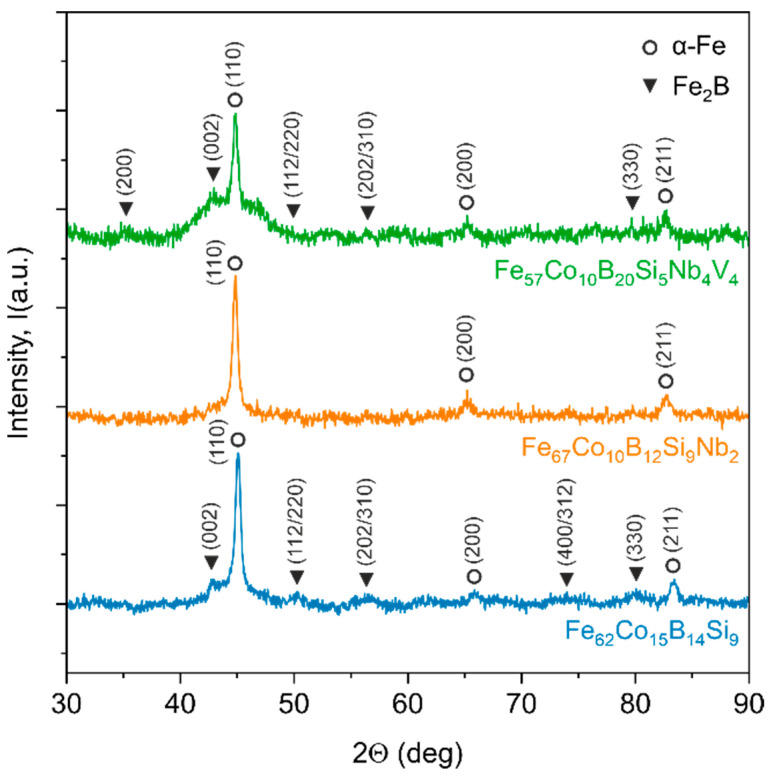
XRD patterns for as-quenched samples.

In order to examine the microstructure of the prepared samples, XRD experiments were conducted, as shown in Figure 4. The diffraction patterns of the as-quenched samples differ from each other. The pattern for the Fe_62_Co_15_B_14_Si_9_ sample consists of crystalline peaks and a slight amorphous halo. A similar tendency in the diffraction pattern can be noticed for the Fe_57_Co_10_B_20_Si_5_Nb_4_V_4_ sample. However, in this case, the amorphous halo is much more visible. In both samples, the characteristic (110), (200), and (211) diffraction peaks correspond to base-centred cubic α-Fe and/or α-FeCo phases. Additionally, (002), (112/220), (202/310), and (330) diffraction peaks for the body-centred tetragonal Fe_2_B phase can be distinguished. The diffraction pattern of the third Fe_67_Co_10_B_12_Si_9_Nb_2_ sample shows only the main diffraction peaks for α-Fe and/or α-FeCo phases. However, the amorphous halo and other diffraction peaks are unnoticeable.

**Figure 5:**
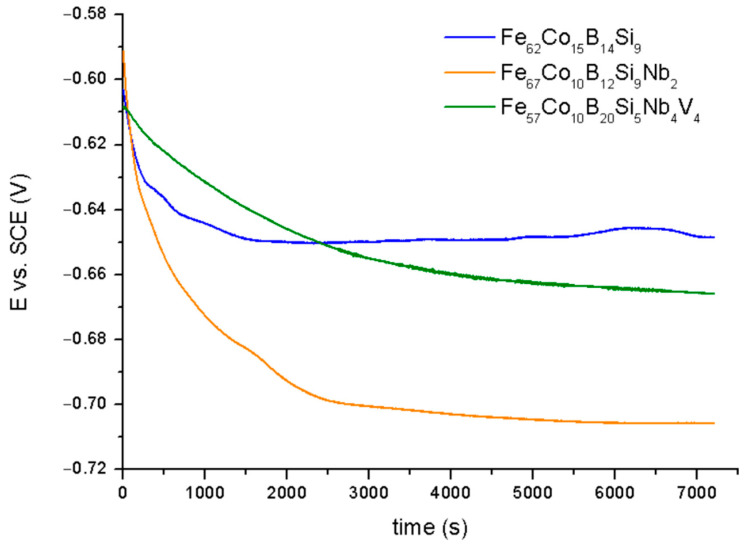
The OCP curves for samples in 0.5 M NaCl.

Figure 5 shows the potential (E_OCP_) in the open circuit potential (OCP) for the Fe_62_Co_15_B_14_Si_9_, Fe_67_Co_10_B_12_Si_9_Nb_2_, and Fe_57_Co_10_B_20_Si_5_Nb_4_V_4_ alloys in 0.5 M NaCl solution.

For all samples, the E_OCP_ was decreased at the beginning of exposure relative to the electrolyte, suggesting that the analysis surfaces absorbed 0.5 M NaCl. Observed behavior was characteristic for materials with the active dissolution of Fe [31]. However, for the Fe_62_Co_15_B_14_Si_9_ sample, after 30 min in immersion, a slight increase in E_OCP_ to –645 mV vs. SCE was observed and this resulted in an increase in the layer consisting of corrosion products on the metallic surface, which is a barrier to the corrosion agent [32]. However, the corrosion product cannot provide effective protection against chloride due to being significantly porous and their composition: Growing iron oxides and iron compounds richer in oxygen, e.g., Fe(OOH)_2_ and FeOOH, creates rust instead of a passivation film [33]. For the Fe_67_Co_10_B_12_Si_9_Nb_2_ sample, the value of E_OCP_ reached −708 mV vs. SCE after 2 h of immersion. A decrease in E_OCP_ for the alloy with the addition of Nb results in the formulation of the thermodynamically unstable oxide form of Nb on their surface [34]. Whereas for the Fe_57_Co_10_B_20_Si_5_Nb_4_V_4_ sample, it stabilized at −660 mV vs. SCE. Increase in the E_OCP_ for the Fe_57_Co_10_B_20_Si_5_Nb_4_V_4_ sample in relation to the Fe_67_Co_10_B_12_Si_9_Nb_2_ sample suggests that addition of V can increase the thermodynamically stability of the alloy in 0.5 M NaCl. According to [35,36], the addition of V decreases the rate of oxygen reduction on work electrode surface. Additionally, a decrease in OCP values for the Fe_67_Co_10_B_12_Si_9_Nb_2_ and Fe_57_Co_10_B_20_Si_5_Nb_4_V_4_ alloys can suggest that the cathodic reaction was limited during the corrosion process [37]. The discrepancy between the samples is most likely due to the diverse chemical nature.

**Figure 6:**
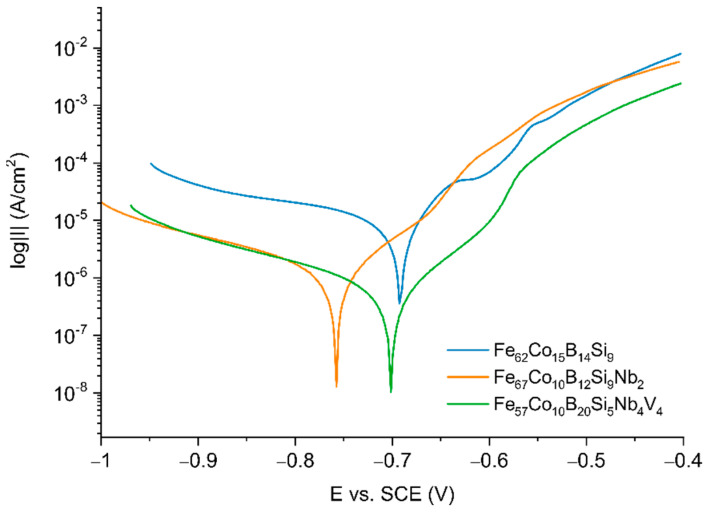
Tafel plots for fabricated alloys.

Figure 6 shows the potentiodynamic polarization behavior of the analyzed samples in 0.5 M NaCl. The corrosion potential (E_corr_) and density corrosion current (j_corr_) were calculated based on the extrapolation of the Tafel region [38].

Electrochemical parameters obtained from polarization were listed in Table 1.

For all samples, active dissolution without any distinctive transition to the passivation region within the applied potential range was observed. The curve for the Fe_62_Co_15_B_14_Si_9_ alloy presented in Figure 6 consists of characteristic regions connected with active dissolution of Fe to near −650 mV, a transition region from −650 mV to −600 mV, and a region before the passivation region near −550 mV, which can be connected with the formation of corrosion products on the alloy surface. It seems to correlate with the processing OCP curves for Fe_62_Co_15_B_14_Si_9_ alloy (Figure 5), where a slight increase in the value of potential during the time of exposition to the electrolyte is observed; this results from the formation of the layer of corrosion products. However, based on the value of characteristic parameters presented in Table 1, it seems that the effect of alloying Nb resulted in a decrease in the kinetics of the anodic dissolution of Fe, which decreased the corrosion current density by four times and increase by one order in the magnitude of resistance polarization for the Fe_67_Co_10_B_12_Si_9_Nb_2_ sample in comparison to the Fe_62_Co_15_B_14_Si_9_ sample. According to the literature, alloying Nb increases the corrosion resistance due to the creation of the passivation film [33]. It seems that the addition of V additionally increases the resistance to corrosion of alloys. For the Fe_57_Co_10_B_20_Si_5_Nb_4_V_4_ sample, a decrease in corrosion current densities (value 1.567 ± 0.941 μA/cm^2^) near eight times was observed. Moreover, a one order in magnitude increase in R_p_ in comparison to Fe_62_Co_15_B_14_Si_9_ alloy was also recorded. The possible anodic dissolution of Fe is shown by Equations (2)–(4) [31].

Fe + (FeOH)_ads_ → Fe(FeOH)_ads_ (2)

Fe(FeOH)_ads_ +OH^−^ → FeOH^+^ + (FeOH)_ads_ +2 e^−^ (3)

FeOH^+^ +H^+^ → Fe^2+^ +H_2_O (4)

For all samples, the B_c_ was lower than B_a_, which suggests that the cathodic reaction connected with reducing oxygen at the interface of the electrolyte/substrate and limited the corrosion [32]. Similar results were received at [32,39] for alloys based on Fe.

**Figure 7:**
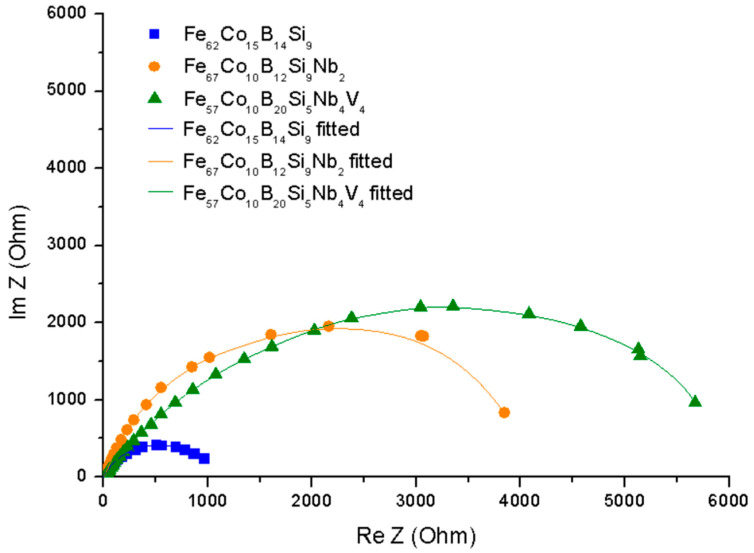
EIS for specimens in 0.5 M NaCl—Nyquist plots.

**Figure 8:**
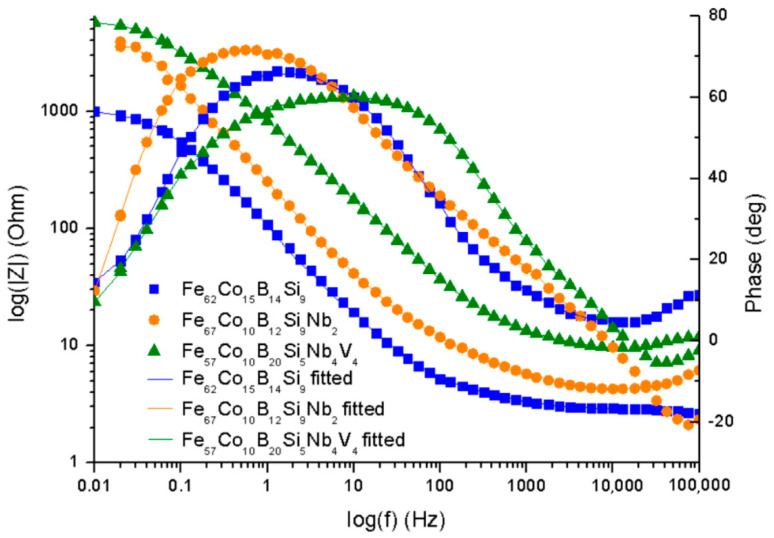
EIS for specimens in 0.5 M NaCl—Bode plots.

**Figure 9:**
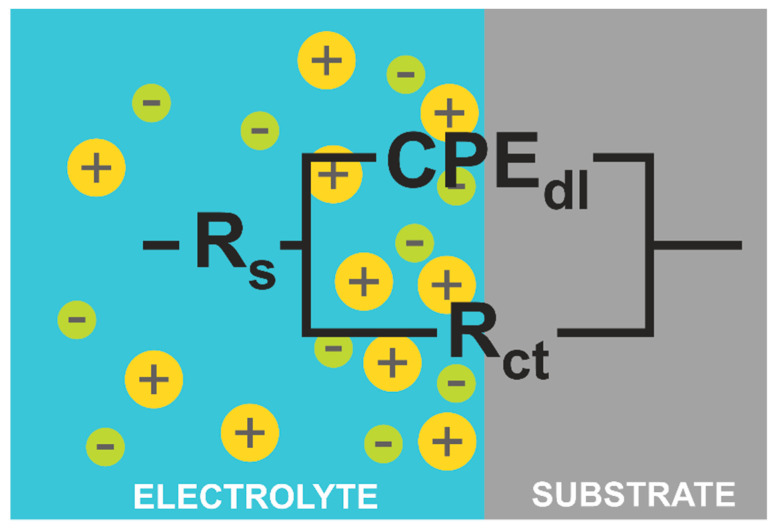
The equivalent electric circuit used to interpret the measured EIS spectra.

The electrochemical impedance spectroscopy was used for the characterization of electrochemical behavior on the electrolyte/substrate interface. Figure 7 shows the Nyquist plot and Figure 8 the Bode plot, both in 0.5 M NaCl. The equivalent electric circuit presented in Figure 9 was used to fit the EIS parameters. The electrical circuit parameters were simulated using the AtlasLab software. The presented circuit equivalents consist of solution resistance (R_s_), a constant phase element connected with a double electric layer (CPE_dl_), and charge transfer resistance (R_st_). Since the capacitor in EIS often does not behave ideally due to the complicated corrosion process at the interface or where the surface is rough or contains inhomogeneities [32], CPE representing a shift from the ideal capacitor is used. The impedance of CPE is defined by Equation (4) [40]:

Z_CPE_ = [Y_o_(jω)^n^]^−1^, (5)

where

- Y_0_—CPE constant;
- j—imaginary number;
- ω—angular frequency (ω = 2πf);
- n—exponent of CPE −1 < n < 1.

For n = −1, the CPE represents an inductor, n = 0 resistor, and n = −1 capacitor. The C_dl_ values can be calculated by Equation (5) [40]:

C = Y_o_(2πf_max_)^n−1^, (6)

where

- f_max_—frequency at which imaginary value reaches a maximum on the Nyquist plot [40].

From the plots obtained (Figure 7 and Figure 8), it can be observed that one loop could be distinguished for the Fe_62_Co_15_B_14_Si_9_, Fe_67_Co_10_B_12_Si_9_Nb_2_, and Fe_57_Co_10_B_20_Si_5_Nb_4_V_4_ alloys. A loop at the high-frequency region appears as a capacitive loop connected with double-layer capacitance parallel to the charge transfer resistance. However, the Nyquist plots (Figure 7) suggest that the Fe_57_Co_10_B_20_Si_5_Nb_4_V_4_ sample proved the higher corrosion resistance. Additionally, a broad loop at 0.1 to 1000 Hz on the Bode plot (Figure 8) was observed for the same alloy, which seems to confirm a higher corrosion resistance and positive influence of Nb and V on corrosion mitigation. Likewise, the loop for the Fe_67_Co_10_B_12_Si_9_Nb_2_ alloy is broader than for the Fe_62_Co_15_B_14_Si_9_ sample, which suggests its higher corrosion resistance. It is believed that alloying of Nb resulted in an increase in corrosion protection. Table 2 shows the characteristic EIS parameters. For the analyzed samples, N_1_ was about 0.72–0.78, which suggests that the CPE was a nonideal capacitor. The presence of a CPE was further confirmed by the phase angle from the Body phase plot (Figure 8). A phase angle of approximately 72° was observed for the Fe_67_Co_10_B_12_Si_9_Nb_2_ alloy and 66° for the Fe_62_Co_15_B_14_Si_9_ alloy. For the Fe_57_Co_10_B_20_Si_5_Nb_4_V_4_ sample, the maximum phase angle was about 60°, indicating a more nonuniform charging and discharging double layer on the surface [41]. However, the CPE values for the Fe_67_Co_10_B_12_Si_9_Nb_2_ sample were about one order in magnitude higher than the Fe_62_Co_15_B_14_Si_9_ and Fe_57_Co_10_B_20_Si_5_Nb_4_V_4_ samples, which can be a result of increased absorption of water in defect during the corrosion process and expanded the capacitance interface [42]. R_ct_ was correlated with the dissolution of Fe and resistance of chargé transfer at the double layer on the electrolyte/substrate interface [43]. Analysis R_ct_ for the Fe_62_Co_15_B_14_Si_9_, Fe_67_Co_10_B_12_Si_9_Nb_2_, and Fe_57_Co_10_B_20_Si_5_Nb_4_V_4_ alloys obtained from fitting the EIS results in 0.5 M NaCl suggests that the addition of Nb and V had a positive influence on the final corrosion protection of fabricated alloys. Alloying Nb and V have influenced a comparatively extended protectiveness of the Fe_57_Co_10_B_20_Si_5_Nb_4_V_4_ alloy in 0.5 M NaCl solution, which is in accordance with literature data [33,44]. Obtained results can be correlated with XRD, where the Fe_67_Co_10_B_12_Si_9_Nb_2_ material shows peaks from crystalline precipitates without the amorphous halo. Increase in the crystalline phase can be severely impaired by the corrosion resistance [33]. The decrease in corrosion resistance of materials with even partial crystallization comes from the fact that any phase segregations and surface heterogeneity, such as various grain orientations, are a potential place for galvanic coupling or instability passive film [45]. α-Fe and Fe_2_B phases influence corrosion deterioration more than crystalline phases rich in Nb [45]. Additionally, the alloying of Nb stabilizes the amorphous phase [46]. For alloys with Nb and V, the observed amorphous halo confirms the amorphous structure of the obtained materials and the decreased share of crystalline phases, which can be a reason to increase the corrosion resistance. What seems to be observed is that not only do the alloying Nb and V induce impact, but also the kind of structure they possess induces a significant impact on the corrosion properties.

**Figure 10:**
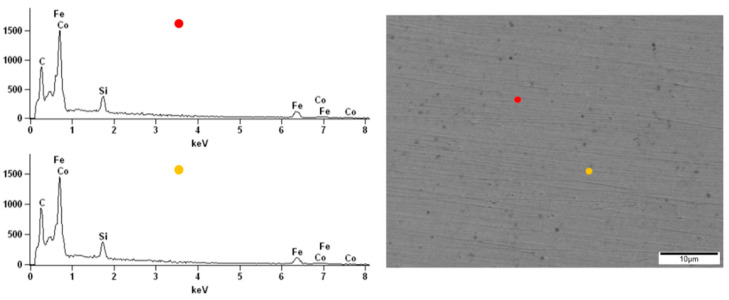
The SEM micrographs of the surface and spectrums of the point EDX analysis for the Fe_62_Co_15_B_14_Si_9_ alloy.

**Figure 11:**
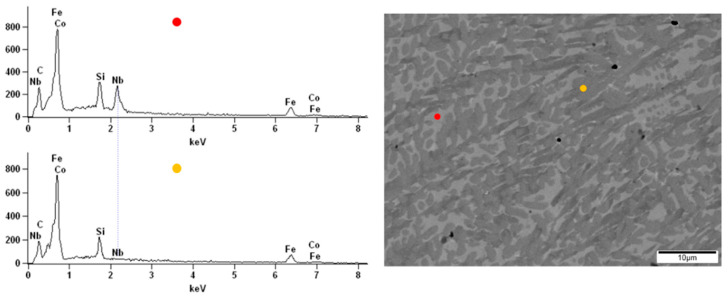
The SEM micrographs of surface and spectrums of the point EDX analysis for Fe_67_Co_10_B_12_Si_9_Nb_2_ alloy.

**Figure 12:**
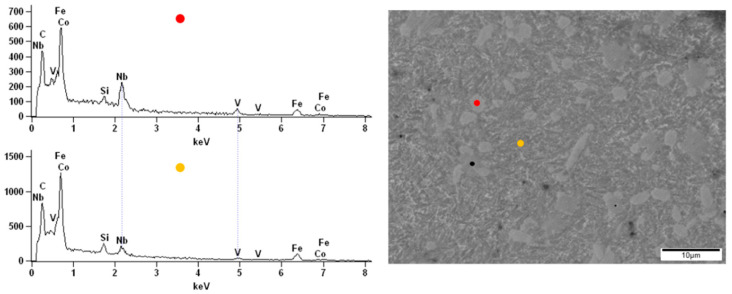
The SEM micrographs of surface and spectrums of the point EDX analysis for the Fe_57_Co_10_B_20_Si_5_Nb_4_V_4_ alloy.

The surface observations of the obtained alloys were carried out by scanning electron microscopy. All samples were analyzed before and after the corrosion test in order to compare the topography changes after exposure to the corrosive environment. The energy-dispersive X-ray spectroscopy (EDX) of the native alloys was performed. Due to the darker and brighter areas observed on the alloy’s surface doped with Nb and Nb/V, we decided to perform point analysis. The surface of the Fe_62_Co_15_B_14_Si_9_ alloy before the corrosion test was homogeneous without any visible structural individuals or divisions (Figure 10). The EDX spectrums obtained from two points on the Fe_62_Co_15_B_14_Si_9_ alloy surface confirmed the homogeneity and consistency of composition. On the native surface of the Fe_67_Co_10_B_12_Si_9_Nb_2_ alloy, the darker and brighter irregular but similar size (few µm) areas were observed (Figure 11). From the point of view of the backscattered electron (BSE) detector used, the aggregation of the Nb in the lighter zones can be assumed. The performed EDX analysis confirmed the presence of the additive element Nb in the alloy and its aggregation in the brighter areas compared to the darker (Figure 11), respectively, 5.53 at.% and 0.23 at.%. In the micrograph of the Fe_57_Co_10_B_20_Si_5_Nb_4_V_4_ alloy surface, the lighter and darker areas are also visible (Figure 12). However, their size was not so uniform as in the case of the Fe_67_Co_10_B_12_Si_9_Nb_2_ alloy. The EDX analysis confirmed the presence of the additive elements Nb and V in the Fe_57_Co_10_B_20_Si_5_Nb_4_V_4_ alloy. There were differences in the proportion of these additives between light and dark areas, i.e., 3.63 at.% and 1.16 at.% for Nb and 3.26 at.% and 1.04 at.% for V.

**Figure 13:**
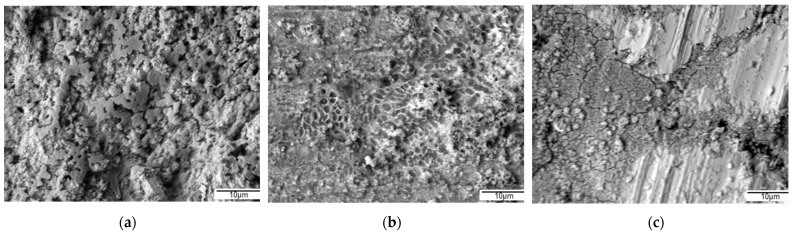
The SEM micrographs of surface of the Fe_62_Co_15_B_14_Si_9_ (**a**), Fe_67_Co_10_B_12_Si_9_Nb_2_ (**b**), and Fe_57_Co_10_B_20_Si_5_Nb_4_V_4_ (**c**) alloys after the corrosion test.

The corrosion processes resulted in the complete covering of the Fe_62_Co_15_B_14_Si_9_ sample surface with a film of corrosion products (Figure 13a). In addition, two levels of the film can be distinguished—the lower continuous layer and the upper one discontinuous layer composed of petals, which was also found in [33]. In the case of the Fe_67_Co_10_B_12_Si_9_Nb_2_ sample, after the corrosion test, the film composed of corrosive products was also observed (Figure 13b). However, compared to the previous Fe_62_Co_15_B_14_Si_9_ material, this film on the Fe_67_Co_10_B_12_Si_9_Nb_2_ alloy is clearly thinner and porous because of the roughness of the substrate that breaks through it. After the corrosion tests, the surface of the Fe_57_Co_10_B_20_Si_5_Nb_4_V_4_ alloy that is only partially covered by corrosion products is observed (Figure 13c). In addition to areas covered with corrosion products, uncovered areas are also visible. Therefore, we can summarize that the greatest surface coverage with corrosion products is observed on the surface of the Fe_62_Co_15_B_14_Si_9_ alloy, less on the Fe_67_Co_10_B_12_Si_9_Nb_2_ alloy, and the least on the Fe_57_Co_10_B_20_Si_5_Nb_4_V_4_ sample.

## 4. Conclusions

Three types of materials were produced based on four alloy components F-B-Co-Si. The primary material with the composition of Fe_62_Co_15_B_14_Si_9_ and two alloys modified by adding alloying elements in the form of Nb (Fe_67_Co_10_B_12_Si_9_Nb_2_) and V (Fe_57_Co_10_B_20_Si_5_Nb_4_V_4_).

The comparison of the microstructure of the tested materials, both with the use of XRD and DSC, showed that the produced alloys have a similar structure and this parameter should not affect their corrosion resistance.

Detailed analysis of corrosion test results shows that the addition of Nb resulted in a decrease in the kinetics of the anodic dissolution of Fe, which decreased the corrosion current densities by four times and increased by one order in the magnitude of resistance polarization for the Fe_67_Co_10_B_12_Si_9_Nb_2_ alloy in comparison to the Fe_62_Co_15_B_14_Si_9_ alloy. Moreover, the addition of V additionally increases the corrosion resistance of the produced materials. For the sample with both addons, which is Fe_57_Co_10_B_20_Si_5_Nb_4_V_4_, a near eight times decrease in corrosion current densities and one order in magnitude increase in resistance polarization in comparison to the Fe_62_Co_15_B_14_Si_9_ base material were observed.

In addition, the increase in corrosion resistance along with subsequent modifications of the layers of materials by adding Nb and V has been confirmed in SEM tests. The microscopic images clearly show the surface changes of the tested alloys after the corrosion tests. It can be summarized that most corrosion products are present on the surface of the Fe_62_Co_15_B_14_Si_9_ alloy, less on the Fe_67_Co_10_B_12_Si_9_Nb_2_ alloy, and the least on the Fe_57_Co_10_B_20_Si_5_Nb_4_V_4_ alloy.

It can be concluded that corrosion tests are of great importance in the case of new materials and small changes in composition can cause significant changes in their resistance to corrosive agents.

Moreover, the results obtained in the presented work may constitute a bridge for application work with the developed materials, particularly in the field of energy harvesting.

## Figures and Tables

**Table 1:** Corrosion performance parameters obtained by potentiodynamic polarization.

Specimen	E_corr_ (mV)	j_corr_ (μA/cm^2^)	B_c_ (mV/dec)	B_a_ (mV/dec)	R_p_ (kΩ cm^2^)
Fe_62_Co_15_B_14_Si_9_	−697.3 ± 8.0	8.743 ± 31.615	82.7 ± 7.3	341.3 ± 75.9	3.608 ± 1.582
Fe_67_Co_10_B_12_Si_9_Nb_2_	−713.8 ± 58.3	2.591± 0.853	105.3 ± 11.0	414.0 ± 93.3	14.627± 4.368
Fe_57_Co_10_B_20_Si_5_Nb_4_V_4_	−683.8 ± 36.9	1.567 ± 0.094	97.6 ± 14.8	226.5 ± 86.5	25.616 ± 8.674

**Table 2:** EIS parameters.

Specimen	Rs (Ω cm^2^)	CPEdL (mF/cm^2^)	N1	Rct (kΩ cm^2^)
Fe_62_Co_15_B_14_Si_9_	10.95 ± 1.44	0.779 ± 0.079	0.73 ± 0.04	1.166 ±0.041
Fe_67_Co_10_B_12_Si_9_Nb_2_	4.76 ± 0.87	1.123 ± 0.162	0.78 ± 0.04	5.792 ± 0.399
Fe_57_Co_10_B_20_Si_5_Nb_4_V_4_	9.77 ± 1.14	0.304 ± 0.136	0.72 ± 0.03	6.691 ± 0.203

## Data Availability

Data supporting reported results can be provided upon request. Currently, these data are collected as part of the ongoing project and only after its completion will the data be made available to the public.
